# *PLoS Biology*—We're Open

**DOI:** 10.1371/journal.pbio.0000034

**Published:** 2003-10-13

**Authors:** Philip Bernstein, Barbara Cohen, Catriona MacCallum, Hemai Parthasarathy, Mark Patterson, Vivian Siegel

## Abstract

With this first issue of PLoS Biology, the editors present the aims and scope of the journal

Welcome to *PLoS Biology*. We would like to introduce you to your journal, one that is run by and for the scientific community in the broadest sense: researchers, teachers, students, physicians, and the public.

One could argue whether scientists need more journals, but we believe there is a global need for greater access to scientific and medical information and that open-access journals can meet this need by removing subscription barriers to the written scientific record. As professional editors, each of us entered the publishing world from the research community with the desire to promote the effective communication and dissemination of science. Offered the opportunity to help spark the transition to open-access publishing by creating an open-access journal that would compete successfully with the most prestigious existing journals, we jumped at the chance.

What you see in this issue is the result of a collaborative effort among the founders, the journal's editorial board, and its professional editors. A glance at the table of contents and the list of outstanding scientists on our editorial board will give you a sense for the scope of the journal, which ranges from molecules to ecosystems and spans the experimental and theoretical disciplines that help to explain our biological world. We aim to publish original articles that address an important question, that challenge our assumptions, that drive science forward.

Our editorial and peer-review process combines the expertise of both professional editors, who are available on a full-time basis to survey the broad landscape of science as well as to engage reviewers and communicate decisions, and academic editors, who understand deeply the strengths and limitations of their area of research. Readers benefit from a selection of exciting and important scientific advances in diverse disciplines. Authors benefit from decisions in which constructive advice is offered and in which papers are not automatically sent back for multiple rounds of review when it is clear to an academic editor who works in the field that concerns raised by reviewers have been satisfied.

Every paper published in *PLoS Biology* is read not only by carefully selected reviewers, but also by an academic editor and a professional editor, who work together throughout the editorial process. Many scientists, both from our editorial board and beyond it, have been our partners in making editorial decisions on the research articles chosen for publication in this and subsequent issues. You will see them listed as academic editors on the individual papers.

Research articles in *PLoS Biology* have no strict length limits; we are keen to provide authors with the opportunity to tell their story in a clear manner and in their own voice. We do not expect, however, that every reader of *PLoS Biology* will be able to appreciate the primary research articles in full, that, for example, a computational neuroscientist will have the specialized vocabulary and working knowledge of an immunologist, or vice versa. What we do expect is that all interested readers will be able to understand and appreciate the Synopses that accompany every article. We also hope that readers will take advantage of our Primers to learn about the tools and topics that are current to the scientific enterprise and of our other magazine content to explore the larger world of science.

We hope that you will lead the open-access revolution by publishing your most exciting research in *PLoS Biology*. And we welcome your ideas for our magazine section. We invite students and scientists at early stages in their careers to present in our Journal Club their critical perspectives and insights on research articles that have captured their imaginations or aroused their skeptical instincts. And we invite other organizations interested in the dissemination and understanding of scientific information to tell us about their activities in our Community Pages.

*PLoS Biology* will be published monthly in print and online. We view our Web site as the primary form of publication for *PLoS Biology,* and we offer different formats to meet the needs of individual readers, such as a version that will help readers with low bandwidth connections and a separate view of the figures and tables in each paper.

The Public Library of Science is committed to making the scientific literature an open resource. The most tangible evidence of this will be the lack of barriers and the ease of navigation that you will experience as you explore our Web site. Many of you with institutional site licenses to other journals might not readily appreciate this ease of access (unless perhaps you are away from your home institution and need that critical paper). But for many of you around the world, it will make the difference between reading the abstract or reading the entire paper. Our goal, however, is not simply to provide a free online journal. Our goal is to create a potent scientific and public resource. As more open-access articles become available, we and many others will be working to develop new tools for integrating, interlinking, organizing, searching, and annotating this expanding collection of information. We invite the community to share these tools as they become available on our site, as well as to propose tools, links, and techniques that would make this treasury of scientific information more useful.

*PLoS Biology* is yours. Download it, copy it, incorporate it in your own database, post or reprint any article; use your imagination. We ask only that you give fair credit to the authors of any work that you use. If you like what you see, e-mail an article to a friend and encourage others to submit their best work. We also invite you to tell us how we can improve the journal and help it evolve into a resource that optimizes the international investment of money, time, energy, and intelligence that goes into the scientific process. You can make a difference. 

